# Supplementary material for: Solar geoengineering could redistribute malaria risk in developing countries
Source: Nat Commun. 2022 Apr 20;13:2150. doi: 10.1038/s41467-022-29613-w (PMC9021229; doi:10.1038/s41467-022-29613-w)

Supplemental Figures

Fig. S1: Populations at risk from *falciparum* malaria in G3 versus RCP 4.5. Transmission risk is split below, into stable risk (left) and unstable risk (right). Populations at risk are reported in millions of people.

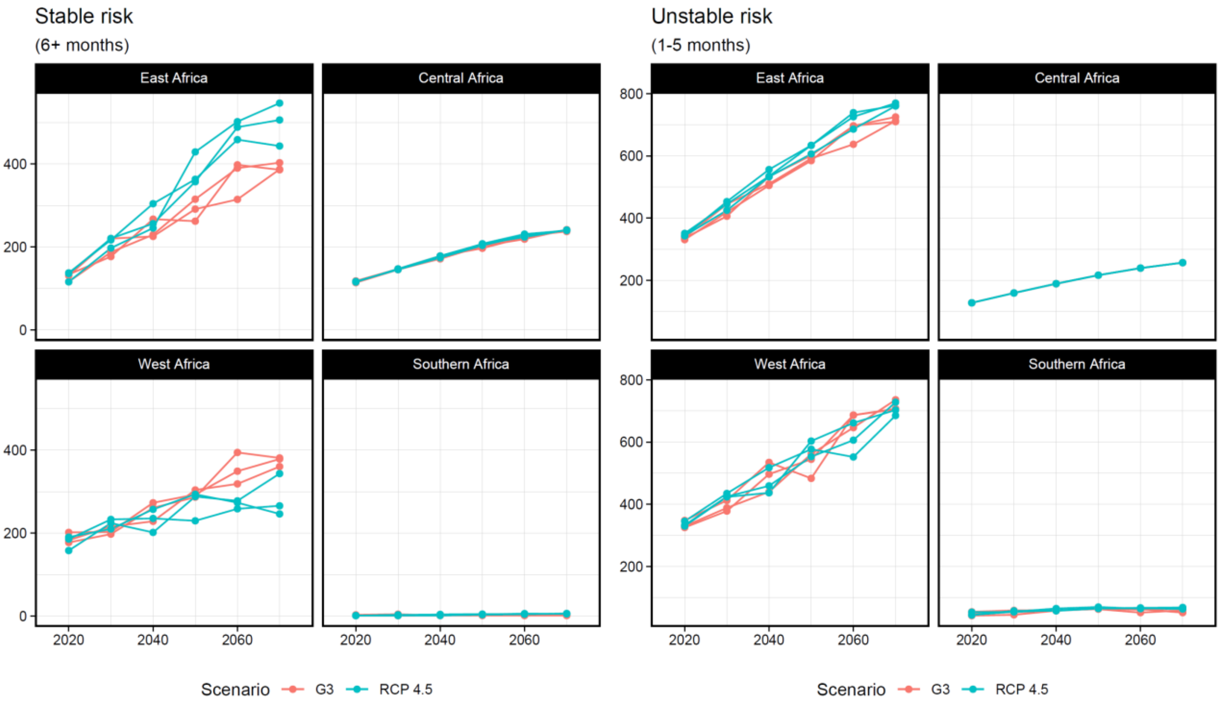

Fig. S2. Populations at risk from *vivax* malaria in RCP 4.5 versus G3. Transmission risk is split below, into stable risk (left) and unstable risk (right). Populations at risk are reported in millions of people.

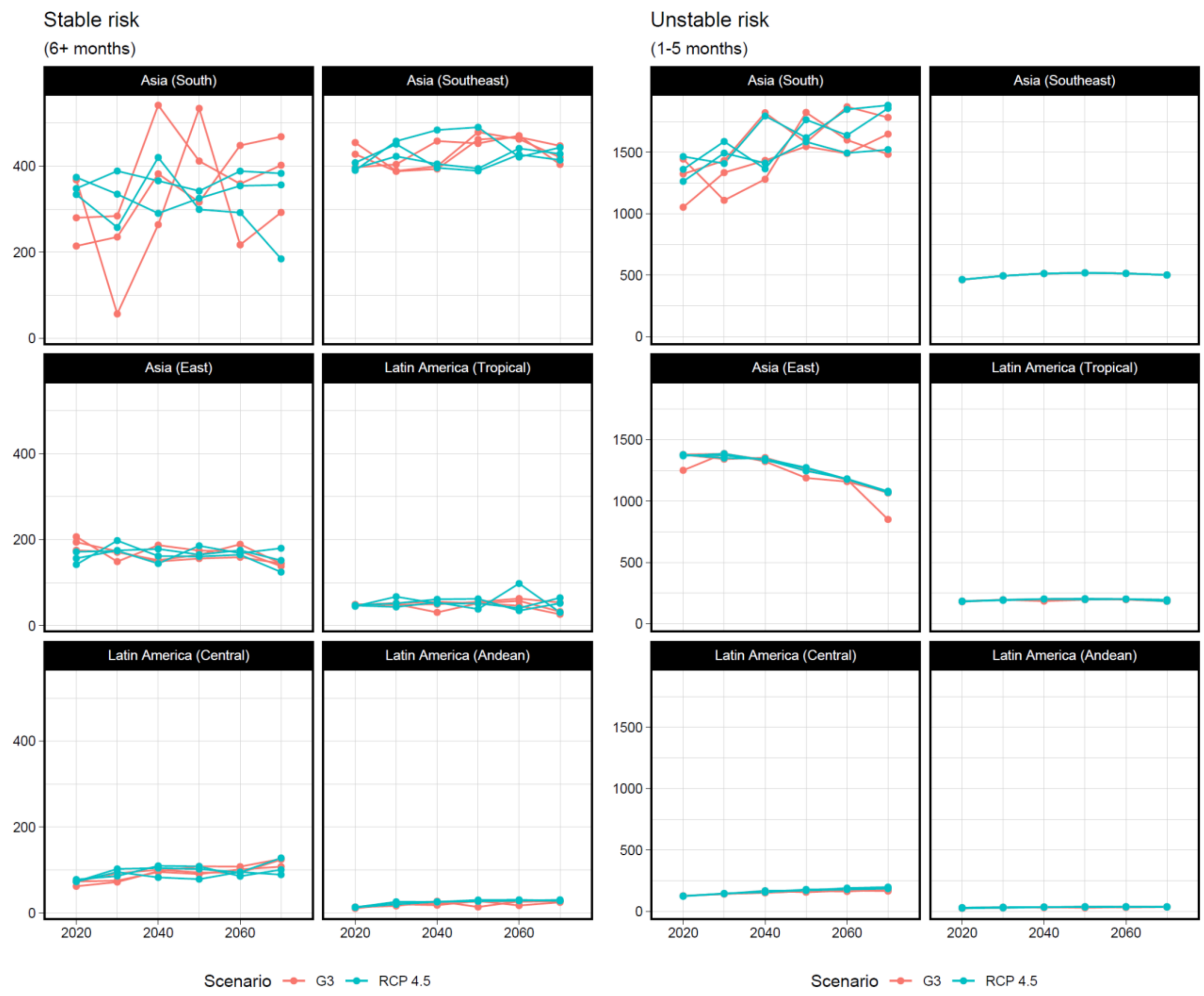

Fig. S3: Populations at risk from *falciparum* malaria in GLENS versus RCP 8.5. Transmission risk is split below, into stable risk (left) and unstable risk (right). Populations at risk are reported in millions of people.

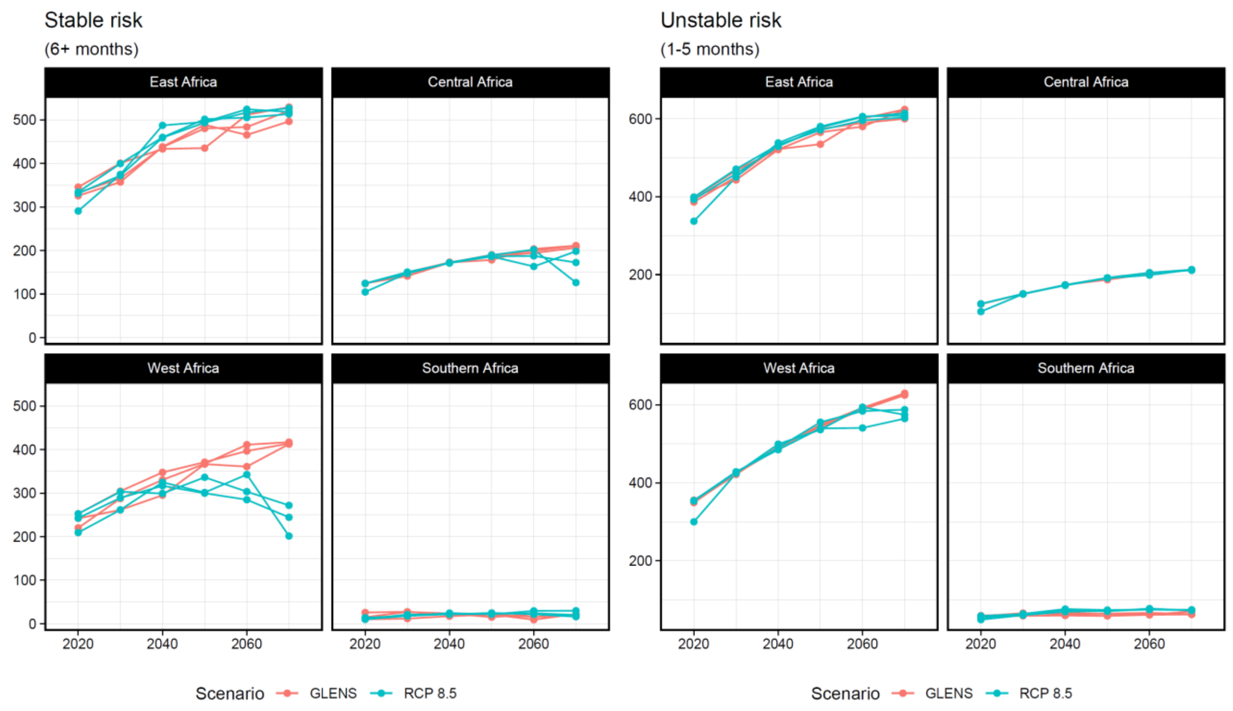

Fig. S4. Populations at risk from *vivax* malaria in RCP 8.5 versus GLENS. Transmission risk is split below, into stable risk (left) and unstable risk (right). Populations at risk are reported in millions of people.

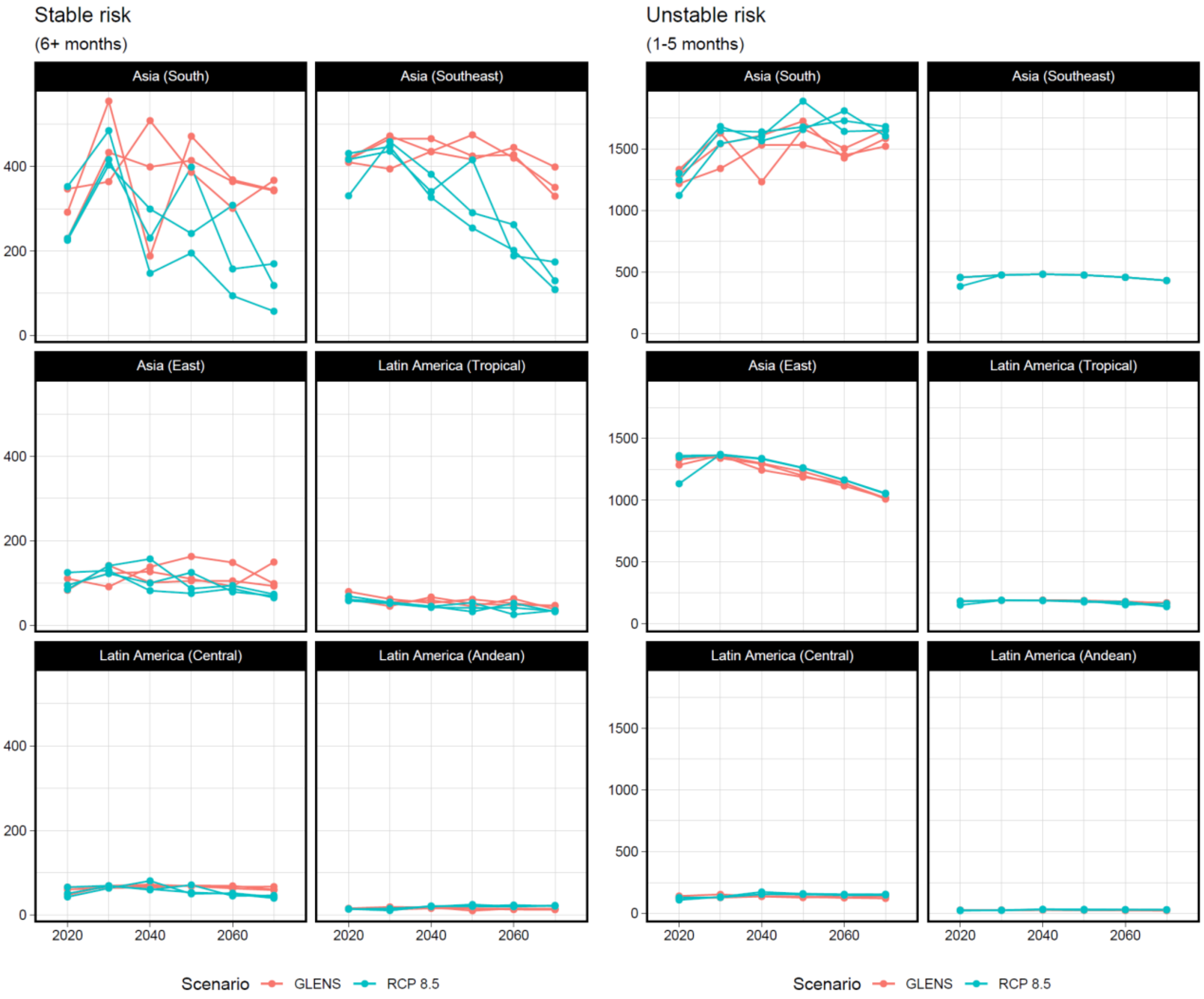

Figure S5. Mean annual temperature in the medium warming scenario. Values are averaged for the years 2020 and 2070.

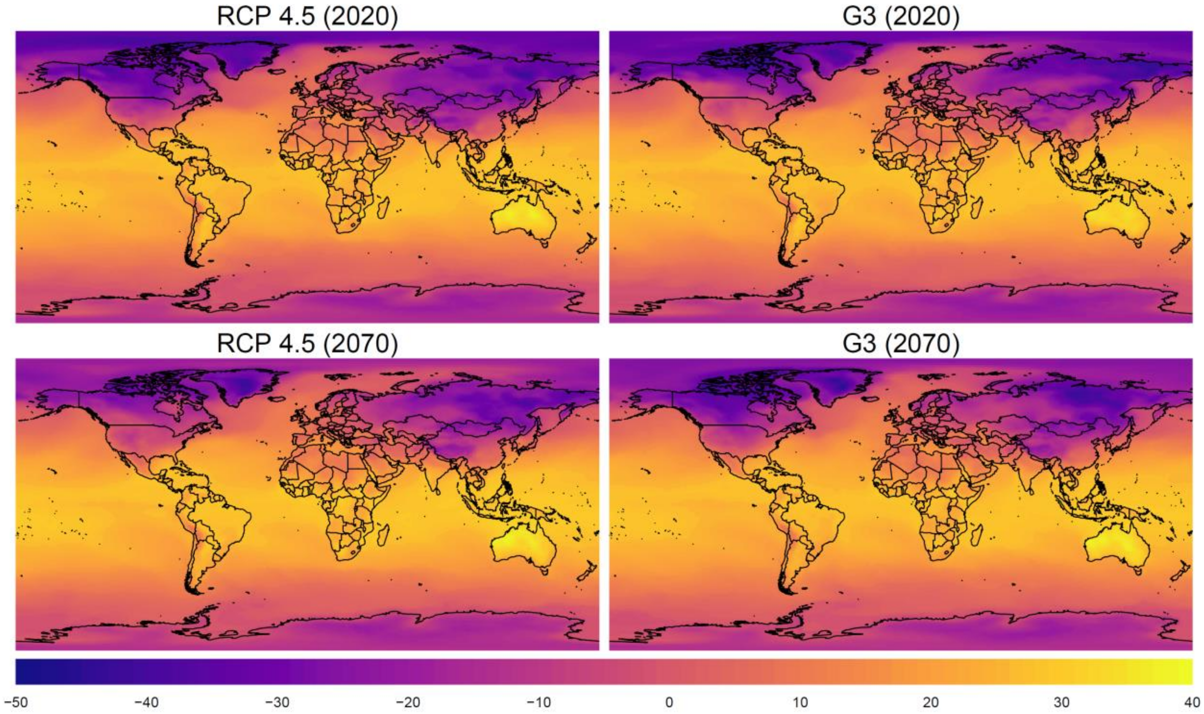

Figure S6. Change in mean annual temperature in the medium warming scenario. Values are averaged for the years 2020 and 2070 and the difference is taken between them.

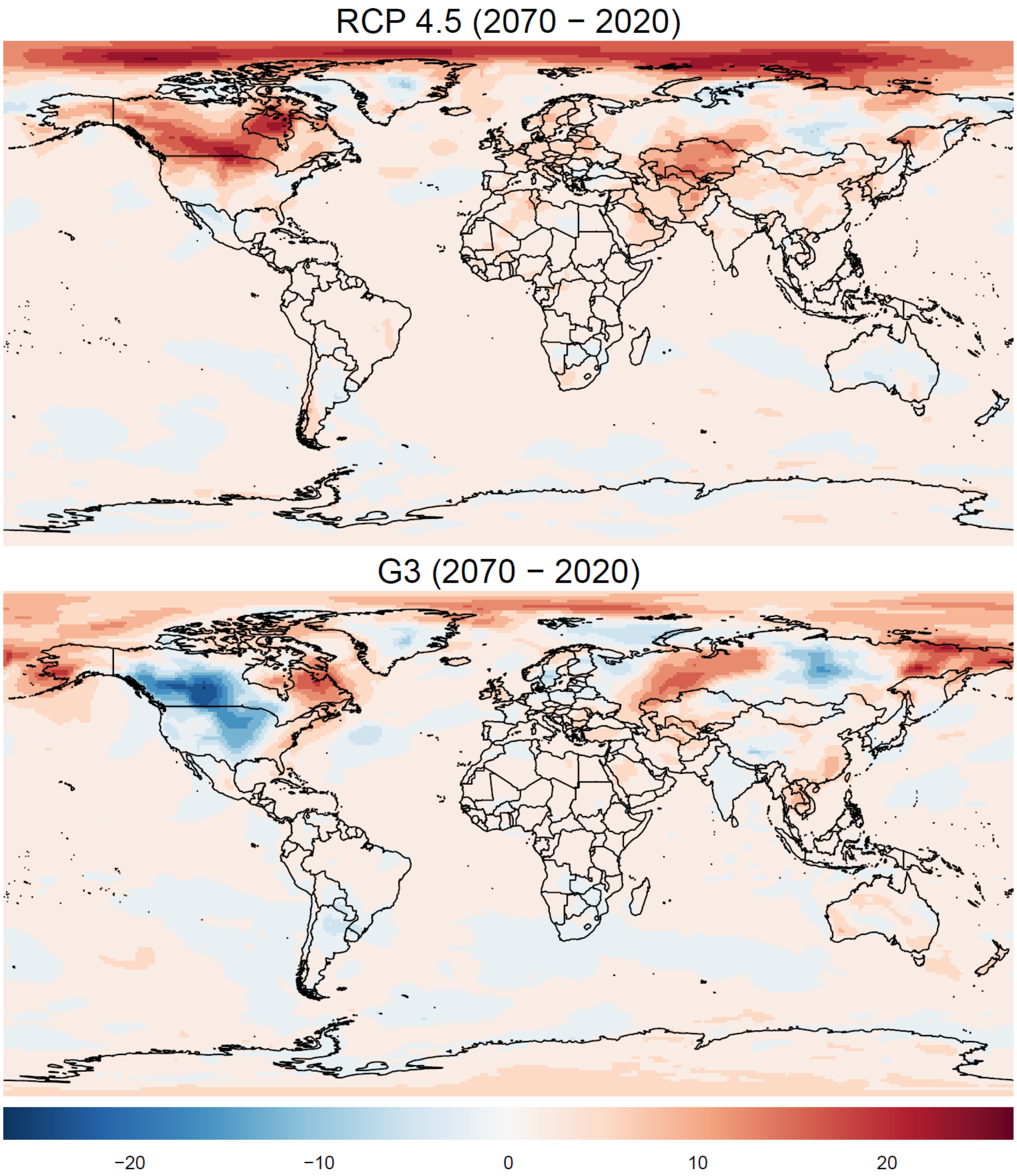

Figure S7. Mean annual temperature in the high warming scenario. Values are averaged for the years 2020 and 2070.

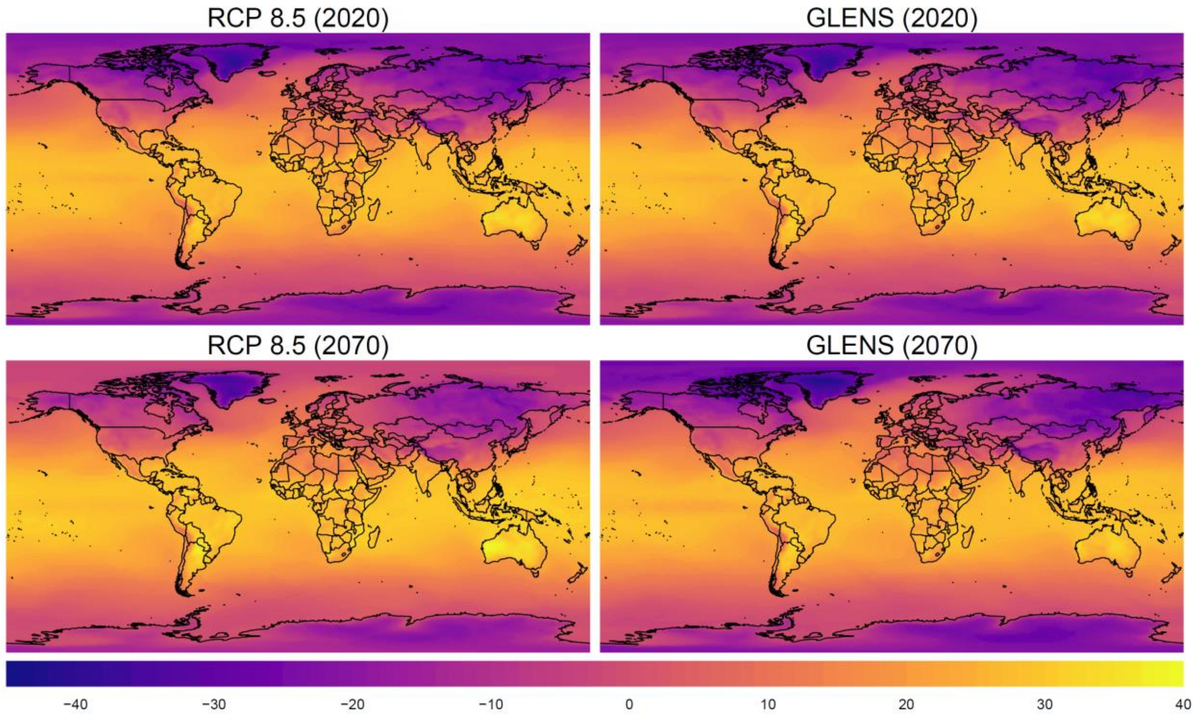

Figure S8. Change in mean annual temperature in the high warming scenario. Values are averaged for the years 2020 and 2070 and the difference is taken between them.

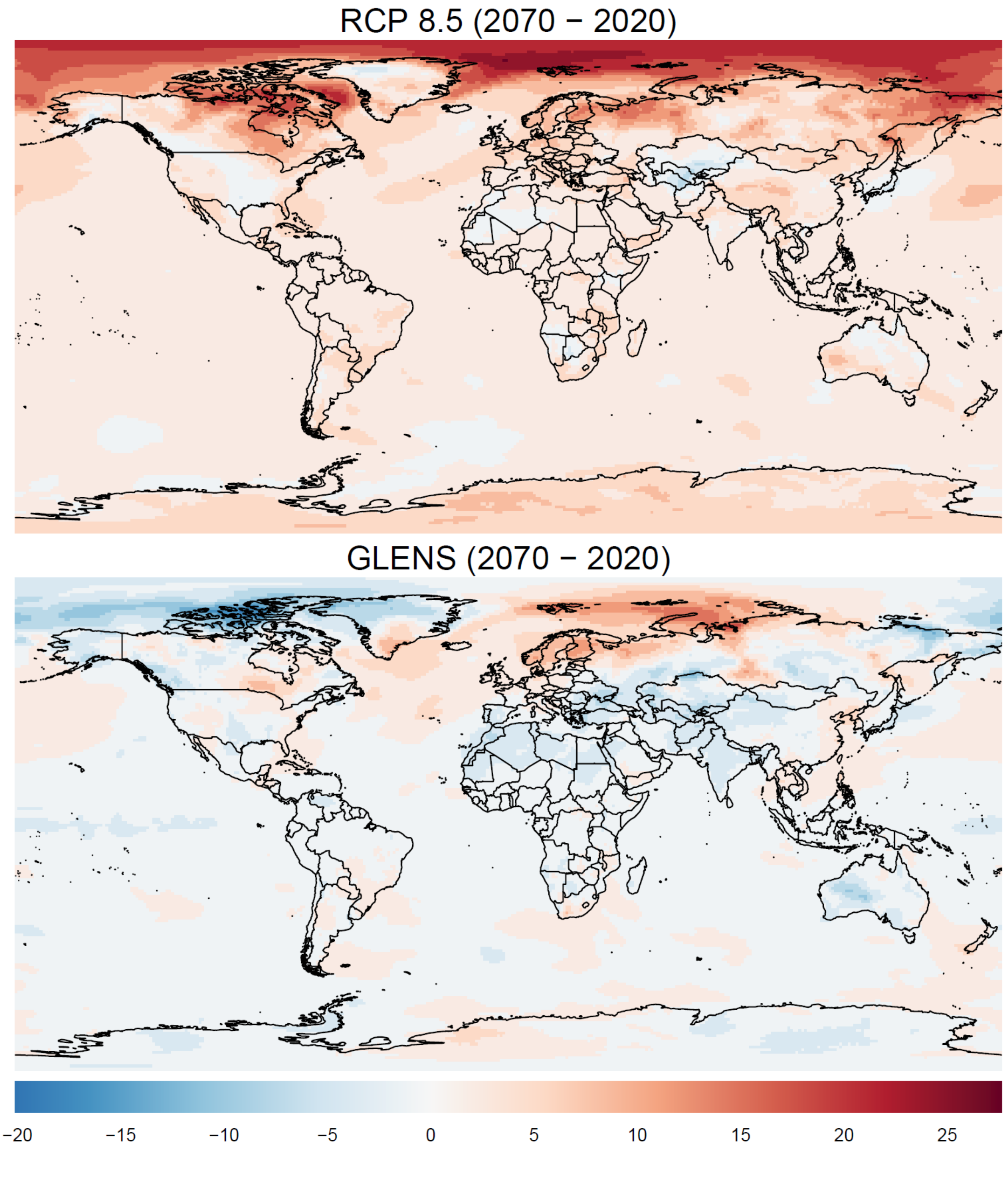

Fig. S9. Recent historical change in *falciparum* malaria prevalence in Africa. Areas with a substantial recent decline in malaria prevalence likely indicate the effect of recent advances in malaria control, and may be the locations where healthcare systems most effectively decouple malaria burden from climate systems, which may be relevant to the future effects of climate change. (A-C) Prevalence values are given based on estimated prevalence of *falciparum* malaria in children aged 2-10 years old (who experience the majority of *falciparum* malaria mortality in Africa). (D) Predicted $R_0(T)$ averaged across RCP 4.5, RCP 8.5, G3, and GLENS scenarios for 2020.

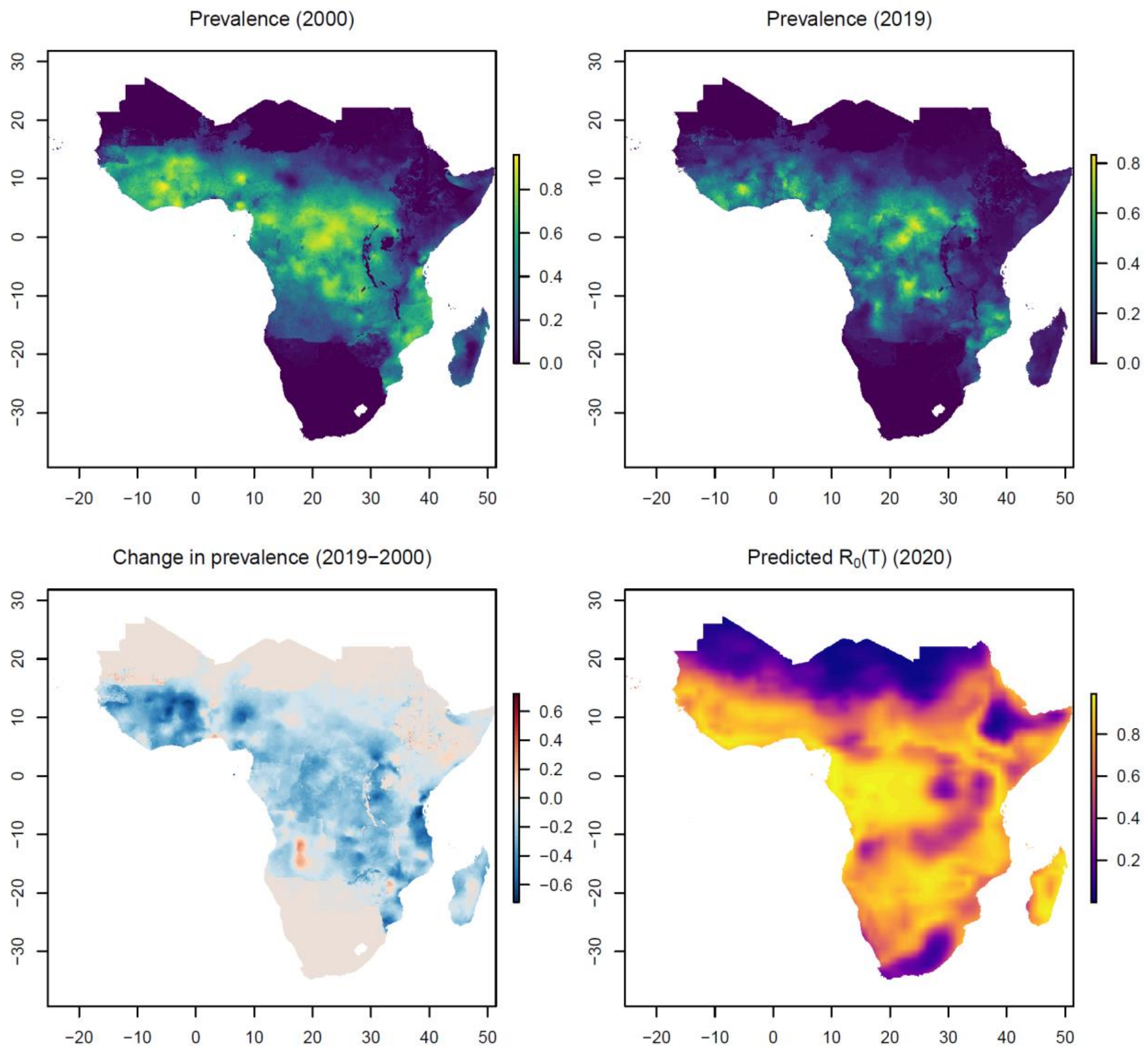

Fig. S10. Recent historical change in *vivax* malaria prevalence in Asia. Areas with a substantial recent decline in malaria prevalence likely indicate the effect of recent advances in malaria control, and may be the locations where healthcare systems most effectively decouple malaria burden from climate systems, which may be relevant to the future effects of climate change. (A-C) Prevalence values are given based on estimated prevalence of *vivax* malaria in the general population (ages 1 to 99 years old). (D) Predicted $R_0(T)$ averaged across RCP 4.5, RCP 8.5, G3, and GLENS scenarios for 2020.

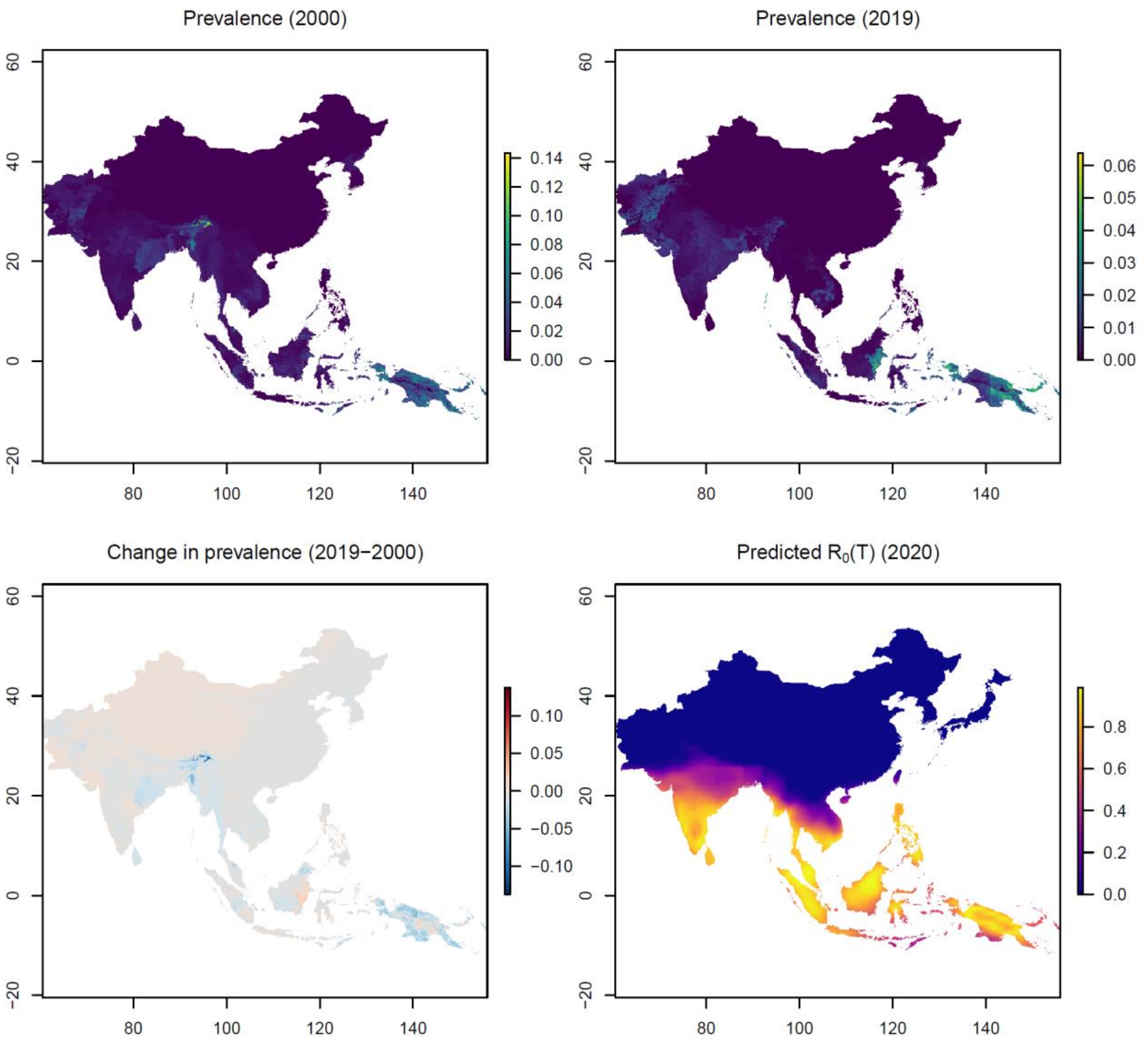

Fig. S11. Recent historical change in *vivax* malaria prevalence in Latin America. Areas with a substantial recent decline in malaria prevalence likely indicate the effect of recent advances in malaria control, and may be the locations where healthcare systems most effectively decouple malaria burden from climate systems, which may be relevant to the future effects of climate change. (A-C) Prevalence values are given based on estimated prevalence of *vivax* malaria in the general population (ages 1 to 99 years old). (D) Predicted $R_0(T)$ averaged across RCP 4.5, RCP 8.5, G3, and GLENS scenarios for 2020.

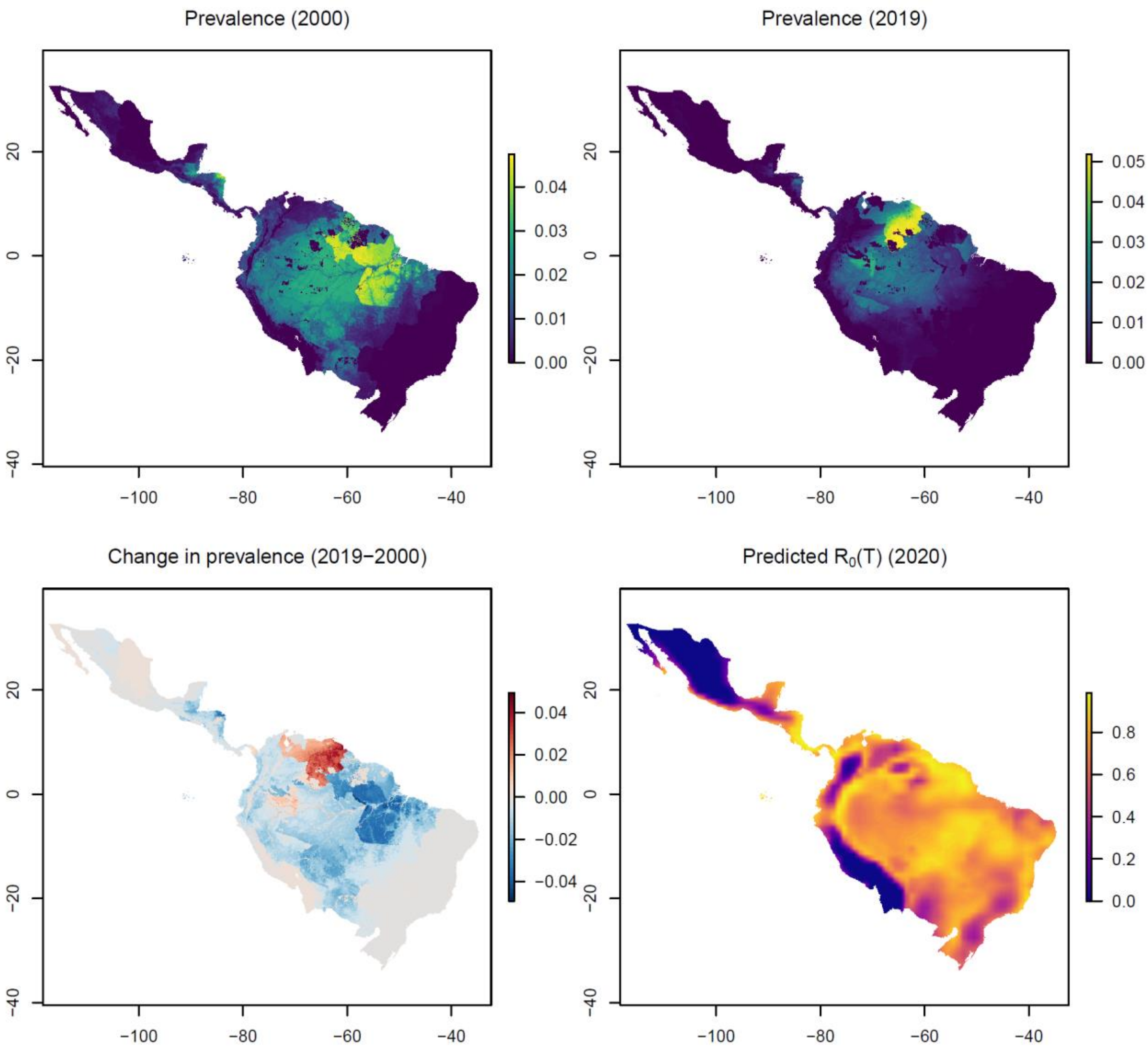

Supplement: Supplementary file 1 — Supplementary Information [file 41467_2022_29613_MOESM1_ESM.pdf]
