# Supplementary material for: Solar geoengineering could redistribute malaria risk in developing countries
Source: Nat Commun. 2022 Apr 20;13:2150. doi: 10.1038/s41467-022-29613-w (PMC9021229; doi:10.1038/s41467-022-29613-w)
Supplement: Supplementary file 3 — Reporting Summary [file 41467_2022_29613_MOESM3_ESM.pdf]

nature portfolio

Corresponding author(s): Carlson
Last updated by author(s): Feb 12, 2022

Reporting Summary

Nature Portfolio wishes to improve the reproducibility of the work that we publish. This form provides structure for consistency and transparency in reporting. For further information on Nature Portfolio policies, see our Editorial Policies and the Editorial Policy Checklist.

Statistics

For all statistical analyses, confirm that the following items are present in the figure legend, table legend, main text, or Methods section.

n/a	Confirmed	
[ ]	[x]	The exact sample size (n) for each experimental group/condition, given as a discrete number and unit of measurement
[x]	[ ]	A statement on whether measurements were taken from distinct samples or whether the same sample was measured repeatedly
[x]	[ ]	The statistical test(s) used AND whether they are one- or two-sided<br>*Only common tests should be described solely by name; describe more complex techniques in the Methods section.*
[ ]	[x]	A description of all covariates tested
[x]	[ ]	A description of any assumptions or corrections, such as tests of normality and adjustment for multiple comparisons
[x]	[ ]	A full description of the statistical parameters including central tendency (e.g. means) or other basic estimates (e.g. regression coefficient) AND variation (e.g. standard deviation) or associated estimates of uncertainty (e.g. confidence intervals)
[x]	[ ]	For null hypothesis testing, the test statistic (e.g. F, t, r) with confidence intervals, effect sizes, degrees of freedom and P value noted<br>*Give P values as exact values whenever suitable.*
[ ]	[x]	For Bayesian analysis, information on the choice of priors and Markov chain Monte Carlo settings
[x]	[ ]	For hierarchical and complex designs, identification of the appropriate level for tests and full reporting of outcomes
[x]	[ ]	Estimates of effect sizes (e.g. Cohen's d, Pearson's r), indicating how they were calculated

Our web collection on statistics for biologists contains articles on many of the points above.

Software and code

Policy information about availability of computer code

Data collection	No novel data were collected for this project.
Data analysis	Code to reproduce the analyses are fully provided in a Github repository (github.com/cjcarlson/geomalaria) which has also been deposited on Zenodo (DOI: 10.5281/zenodo.6054510).

For manuscripts utilizing custom algorithms or software that are central to the research but not yet described in published literature, software must be made available to editors and reviewers. We strongly encourage code deposition in a community repository (e.g. GitHub). See the Nature Portfolio guidelines for submitting code & software for further information.

Data

Policy information about availability of data

All manuscripts must include a data availability statement. This statement should provide the following information, where applicable:
- Accession codes, unique identifiers, or web links for publicly available datasets
- A description of any restrictions on data availability
- For clinical datasets or third party data, please ensure that the statement adheres to our policy

Smaller datasets to reproduce the study - the population grids, Malaria Atlas Project files, and thermal suitability curve estimates - are available on the Github repository (github.com/cjcarlson/geomalaria). All of the climate data are deposited on Dryad at DOI: 10.5061/dryad.7m0cfxpwg

Field-specific reporting

Please select the one below that is the best fit for your research. If you are not sure, read the appropriate sections before making your selection.

☐ Life sciences ☐ Behavioural & social sciences ☒ Ecological, evolutionary & environmental sciences

For a reference copy of the document with all sections, see nature.com/documents/nr-reporting-summary-flat.pdf

Ecological, evolutionary & environmental sciences study design

All studies must disclose on these points even when the disclosure is negative.

Study description	We project the possible population at risk and transmission efficacy of malaria around the world in future climate scenarios with and without the deployment of solar geoengineering.
Research sample	We use global climate models that simulate the world with and without solar geoengineering.
Sampling strategy	Not applicable (no sampling to collect the data).
Data collection	Not applicable (no primary data collected or generated in this study).
Timing and spatial scale	Global, over the 21st Century.
Data exclusions	Not applicable (no data excluded).
Reproducibility	Our analysis is fully reproducible with the files on the Dryad and Github repositories, and has been locally reproduced to ensure final results are accurate.
Randomization	Not applicable (no experimental trials undertaken in the study; only deterministic simulations).
Blinding	Not applicable (no experimental trials undertaken in the study; only deterministic simulations).

Did the study involve field work? ☐ Yes ☒ No

Reporting for specific materials, systems and methods

We require information from authors about some types of materials, experimental systems and methods used in many studies. Here, indicate whether each material, system or method listed is relevant to your study. If you are not sure if a list item applies to your research, read the appropriate section before selecting a response.

Materials & experimental systems

n/a	Involved in the study	
☒	☐	Antibodies
☒	☐	Eukaryotic cell lines
☒	☐	Palaeontology and archaeology
☒	☐	Animals and other organisms
☒	☐	Human research participants
☒	☐	Clinical data
☒	☐	Dual use research of concern

Methods

n/a	Involved in the study	
☒	☐	ChIP-seq
☒	☐	Flow cytometry
☒	☐	MRI-based neuroimaging
